# A Mini Review of Antibacterial Properties of Al_2_O_3_ Nanoparticles

**DOI:** 10.3390/nano12152635

**Published:** 2022-07-30

**Authors:** Sergey V. Gudkov, Dmitriy E. Burmistrov, Veronika V. Smirnova, Anastasia A. Semenova, Andrey B. Lisitsyn

**Affiliations:** 1 Prokhorov General Physics Institute of the Russian Academy of Sciences, 38 Vavilova St., 119991 Moscow, Russia; dmitriiburmistroff@gmail.com (D.E.B.); veronausckova@mail.ru (V.V.S.); 2 V.M. Gorbatov Federal Research Center for Food Systems of the Russian Academy of Sciences, 109316 Moscow, Russia; a.semenova@fncps.ru (A.A.S.); info@fncps.ru (A.B.L.)

**Keywords:** aluminum oxide, nanoparticles, antibiotic resistance, antibacterial, cytotoxicity, bacteriostatic, antibacterial effect

## Abstract

Bacterial antibiotic resistance is one of the most serious modern biomedical problems that prioritizes the search for new agents to combat bacterial pathogens. It is known that nanoparticles of many metals and metal oxides can have an antibacterial effect. However, the antibacterial efficacy of aluminum oxide nanoparticles has been studied little compared to the well-known antimicrobial properties of nanoparticles of oxides of metals such as zinc, silver, iron, and copper. In this review, we have focused on the experimental studies accumulated to date demonstrating the antibacterial effect of aluminum oxide nanoparticles. The review discusses the main ways of synthesis and modification of these nanoparticles, provides the proposed mechanisms of their antibacterial action against gram-positive and gram-negative bacteria, and also compares the antibacterial efficacy depending on morphological characteristics. We have also partially considered the activity of aluminum oxide nanoparticles against water microalgae and fungi. In general, a more detailed study of the antibacterial properties of aluminum oxide nanoparticles is of great interest due to their low toxicity to eukaryotic cells.

## 1. Introduction

Metal oxide nanoparticles (NPs) are popular and inexpensive in production materials that have found an increasing application in modern life due to their unique properties. The global market of nanometals based on metal oxides was estimated at a level of USD 4.2 billion in 2016. By 2025, a growth in the demand for NPs production is forecast, which is conditioned by extensive research carried out in the biomedical sector using materials based on metal oxide NPs [1]. In 2020, the number of publications (more than 400) and patents (about 200) regarding the use of metal oxide NPs as a therapeutic tool and antibacterial agents found in Scopus was twice as high as that in 2015. Particular attention has been given to studies aimed at the possibility of using nanoparticles as biosensors [2], for diagnosis and therapy of oncological diseases [3], and for drug delivery [4]. The use of nanomaterials based on metal oxide nanoparticles to control bacterial infections [5,6,7] including antibiotic resistant [8] is of great interest. Nowadays, there are many studies demonstrating the antibacterial effect of zinc oxide [9], iron oxide [10], titanium dioxide [11], silver oxide [12], copper oxide [13], and other nanoparticles. Aluminum oxide nanoparticles (AlOxNPs) are the other interesting candidate. It is known that these particles do not have pronounced cytotoxicity due to inertness of aluminum oxide [14,15]; nevertheless, a question about the antibacterial properties of these nanomaterials is open for discussions and requires more detailed investigation.

Aluminum is the most abundant element in the Earth’s crust (~8%) and the third most abundant element in the composition of the lithosphere. As is well known, aluminum does not take part in important biological processes. Although all modern living organisms contain some amounts of aluminum, there is no scientific evidence of aluminum participation in normal biochemical processes in organisms. Moreover, any proof of a role of aluminum in biochemical processes in organisms over the course of evolution is also absent. As a result, a lack of the biological role of aluminum on the background of its abundance remains to be a kind of “biochemical puzzle” [16].

Aluminum is an active amphoteric metal and in the normal conditions forms a white oxide film on the surface. The most well-known phase modifications of aluminum oxide are α-, β-, and γ- Al_2_O_3_. In nature, the most commonly occurring modification is α-modification of aluminum oxide (α-Al_2_O_3_) also known as alumina, which with silica is a basis of clay-forming minerals. Pure Al_2_O_3_ occurs as the mineral corundum and its rare varieties (ruby, sapphire and so on). α-Al_2_O_3_ is used as an abrasive material, a raw material for production of pure aluminum, as well as for production of fireproof materials because of its high melting temperature. Crystals from corundum varieties are working bodies of lasers; stones for precise mechanisms are made from rubies. This phase is the only thermodynamically stable form of Al_2_O_3_.

Upon heat treatment of aluminum hydroxides at about 400 °C, γ-form of aluminum oxide is obtained. γ-Al_2_O_3_ is used as a carrier of catalyzers and a desiccant in processes of chemical and petrochemical production. Heating up to 1100–1200 °C facilitates irreversible transformation of the γ-modification into α-Al_2_O_3_ [17]. β-aluminum oxide has a hexagonal crystal lattice. β-Al_2_O_3_ is not a true aluminum oxide but is a mixture of aluminates of alkali and alkaline earth metals with the high content of aluminum oxide. At temperatures of 1600–1700 °C, the β-modification breaks down into α-Al_2_O_3_ and the corresponding metal oxide, which is discharged as a vapor. There is also amorphous aluminum oxide, alumogel, formed upon desiccation of gel-like Al(OH)_3_ and representing a porous and sometimes transparent substance. Alumogel is widely used in technique and medicine as an adsorbent.

Nanosized aluminum oxide (α-and γ- Al_2_O_3_) has found an increasing application in various fields due to its unique properties, such as the high mechanical strength, large surface area in reference to the volume, high firmness, and good chemical stability [18,19]. In particular, it is proposed to use AlOxNPs as catalyzers [20], adsorbents [21], additives to concrete mixtures [22], tribological additives for lubricating liquids, raw materials for ceramic production [23], in cosmetic and textile industries [24], as well as in microelectronics [25]. A possibility of using AlOxNPs with the biomedical purposes [26,27], in particular, as an antibacterial agent, is of great interest; today, however, there are few data about mechanisms of action of these nanoparticles on the microbial growth.

This review focuses on the literature data about the antibacterial properties of AlOxNPs, discusses the main ways of synthesis of these nanoparticles and the possible solutions for increasing their antibacterial activity, and presents the analysis of the research results accumulated up to date that are relevant to the effect of AlOxNPs on microbiological objects.

## 2. Literature Review

### 2.1. Process of Searching Articles

A search for publications was carried out using several search services (Google Scholar, Web of Science, and Scopus). In searching for papers, the tags “antibacterial”, “nanoparticles”, “aluminum oxide”, “Al_2_O_3_”, and “antimicrobial” were used in different combinations. When publications were found, we did not sample particular papers but considered each paper presented by the search engine. Thus, we found 37 papers devoted to the study of the action of aluminum oxide nanoparticles of microbiological objects, mainly on bacterial cells. Then, we constructed a table containing the brief information for each of the found papers by the following categories: a NP synthesis method, size, form, used concentration, medium and conditions of microbial cultivation, tested bacteria, and biological effect (see Table 1).

### 2.2. Ways of Synthesis and Possible Methods for Improving AlOxNP Properties

Various approaches for AlOxNP synthesis are used including bottom-up and top- down methods. The most used top-down methods are laser ablation [28,29] and ball milling [30]. Other methods include sol–gel process [31], microemulsion method [32], microwave processing, [19,33,34], solvothermal synthesis [35], and combustion [36]. Laser ablation is a widely used method for NP production, which allows to perform synthesis in different media: in vacuum, liquid, and gas. The advantages of this method are high rate of the synthesis process, purity of a synthesized product, and a possibility to adjust finely characteristics of obtained nanomaterials [28].

**Figure 1:**
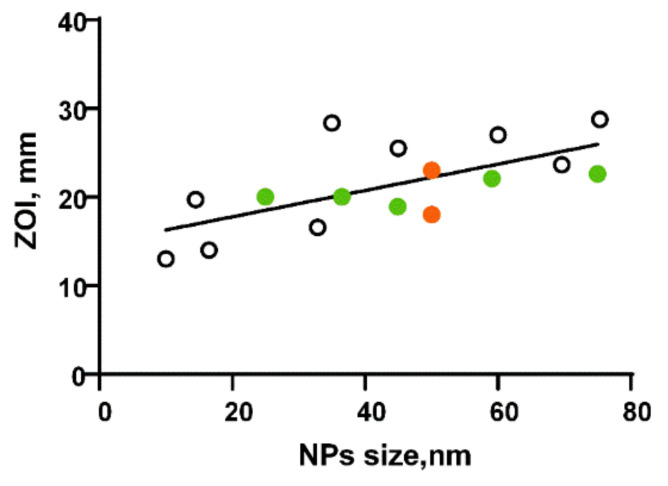
Dependence of the inhibition zone on the AlOxNP size reported in the literature for *S. aureus*. Green dots-NPs, synthesized using plant extracts; orange dots-NPs, modified with chitosan.

The chemical precipitation method [37] and microwave heating [19,38] are also widely used for synthesis of metal oxide nanoparticles, including AlOxNPs. The chemical precipitation method is simple, cost-efficient, and does not require high-technology equipment [39]. Special attention is given to the AlOxNP “green synthesis” methods including the use of plant extracts during AlOxNP chemical synthesis, generally, as a reducing agent. In particular, a successful use of extracts of *Prunus* × *yedoensis* [19], *L. majucula* [40], *Colletotrichum* sp. [34], *Urtica dioica* [41], and *Cymbopogon citratus* [38,42] was noted in the AlOxNP synthesis. However, the use of plant extracts in AlOxNP synthesis did not lead to an increase in the antibacterial effect (Figure 1). Several papers reported about high effectiveness of nanocomposite materials with nanoparticles of other metals and metal oxides as well as with the use of polymers containing AlOxNPs in the composition. For example, Al_2_O_3_–Ag composite showed the bacteriostatic activity against both *E. coli* and *S. epidermidis*; with that, an effect against *E. coli* was not observed when using pure AlOxNPs [43].

**Figure 2:**
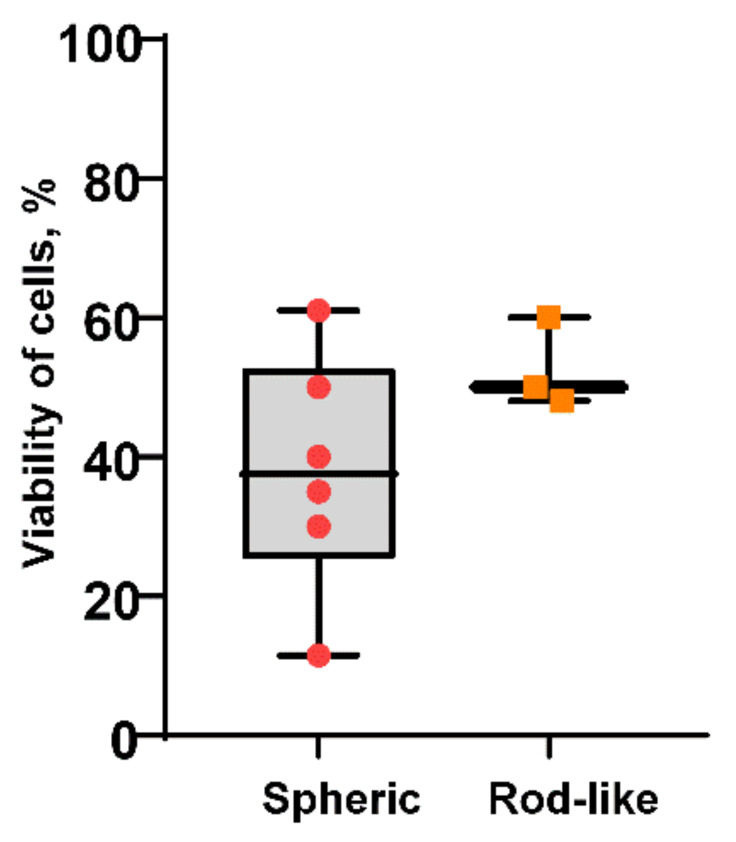
Comparison of the antibacterial effectiveness of AlOxNPs with spherical and rod-like morphology reported in literature.

The use of biodegradable polymers, such as polylactide (PLA), polyglycolide (GLA), their copolymer (PLGA), alginic acid, gelatin, and others together with metal oxide nanoparticles, including AlOxNPs, is a promising approach to increasing both the biocompatibility and antibacterial properties of materials. Several studies considered AlOxNP modification with chitosan, which allowed enhancing the antibacterial properties of materials under consideration [44,45,46]. Yakumi et al. [47] constructed PLA-based nanocomposites that contained Al_2_O_3_ and TiO_2_ as a filler (PLA/Al_2_O_3_ and PLA/TiO_2_-Al_2_O_3_). The obtained composite materials inhibited the growth of *P. aeruginosa* and *E. coli*. An increase in the nanoparticle concentration in the formulation of composite materials facilitated an increase in their bacteriostatic properties. A higher effectiveness of growth inhibition of the tested bacteria was observed when using PLA/TiO_2_-Al_2_O_3_ compared to PLA/Al_2_O_3_. Therefore, the examined methods for AlOxNP modification make it possible to enhance the antimicrobial potential of these nanoparticles by creating composite materials both by addition of nanoparticles with the high bactericidal activity and by the use of polymer materials. AlOxNPs are obtained in three main forms: spherical, rod-like, and flake-like. Based on the found literature data, it was established that synthesized AlOxNPs had mainly a spherical morphology (*n* = 21). In addition, rod-like AlOxNPs were obtained in three analyzed studies, while the antibacterial properties of flake-like nanoparticles were investigated only in one study. We compared the antibacterial effect of two morphological varieties of AlOxNPs (spherical and rod-like); however, statistically significant difference was not revealed due to the low number of elements of sampling (Figure 2).

### 2.3. Peculiarities of Action of Aluminum Oxide Nanoparticles against Bacterial Cells

AlOxNPs exert a significant effect of the growth of bacterial cultures in vitro, as a rule, at high concentrations (≥1000 µg/mL) (see Table 1). The action is mainly characterized by retardation of the colony growth and an increase in inhibition zones; that is, the bacteriostatic effect is exerted. AlOxNPs did not show significant toxicity to common soil bacteria *Bacillus cereus* and *Pseudomonas stutzeri* [48]. Several studies reported about a moderate bacteriostatic effect of aluminum oxide nanoparticles at a concentration of 1 mg/mL and a size of 180 nm against *E. coli* [49,50]. A 40% decrease in the growth rate of *P. putida* bacterial cultures upon AlOxNP addition was observed compared to the application of non-nanosized Al_2_O_3_ [51]. It is also important to note that several studies reported about the inhibitory effect against multiresistant Gram-negative and Gram-positive bacteria as well as clinical strains. In particular, they demonstrated a decrease in the growth rate of *S*. *aureus* ATCC 25923, MSSA, and MRSA strains by about 8 times upon AlOxNP addition at a concentration of 1000 µg/mL and by about 16 times at a concentration of 2000 µg/mL after 16 h of exposure [52]. Moreover, the inhibitory and bactericidal action of AlOxNPs against multiresistant clinical isolates of *P. aeruginosa* was reported. MIC and MBC ranges of 1600–3200 µg/mL and 3200–6400 µg/mL, respectively, were recorded [38]. In another study, the same authors also examined the influence of AlOxNPs on the growth of multiresistant clinical isolates of *E. coli.* MIC and MBC ranges corresponded to those reported for *P. aeruginosa* [50]. The inhibitory action of AlOxNPs at moderate concentrations and bactericidal action at high concentrations were also revealed for multiresistant strains of *A. baumanii*. The MIC and MBC range was 125 to 1000 µg/mL [53].

A number of works have compared the antibacterial effect of AlOxNPs with nanoparticles of oxides of other metals. For example, Sikora et al. [54] examined the efficacy of nanomaterials based on Al_2_O_3_, CuO, Fe_3_O_4_, and ZnO against *S. aureus*, *P. Aeruginosa*, *C. albicans*, and 4 *E. coli* strains (ATCC^®®^8739™, MG1655, MDS42, and MDS69). All considered samples of nanoparticles inhibited the growth of microorganisms. Fe_3_O_4_ nanoparticles exerted the greatest inhibitory effect on *E. coli* ATCC^®®^8739™, and ZnO nanoparticles on *E. coli* MG1655. Overall, in both the 4 h acute toxicity test and the 24 h experiment, ZnO nanoparticles had the highest antibacterial potential and AlOxNPs the lowest. Interestingly, AlOxNPs inhibited the growth of *E. coli* more effectively than other nanomaterials [54]. Manyasree et al. [55] also compared the efficacy of AlOxNPs, CuO, Fe_3_O_4_, and ZnO at various concentrations (10–50 µg/mL) against *E. coli*, *P. vulgaris*, *S. aureus*, and *S. mutans*. At the same time, a high sensitivity of *E. coli* to Al_2_O_3_ nanoparticles was observed, which is in good agreement with the previous report; CuO-NPs were effective against *P. vulgaris* and *S. mutans*, and Fe_2_O_3_ against *S. aureus* [55].

The phase composition of AlOxNPs can be an important factor determining the antibacterial properties of these nanomaterials. Pakrashi et al. [56] demonstrated a higher antibacterial activity of γ-phase of aluminum oxide compared to α- aluminum oxide against *Bacillus licheniformis* after two-hour exposure, which was manifested in a higher content of ROS after exposure to γ-Al_2_O_3_ (2.6 ± 0.02%) compared to α-Al_2_O_3_ (0.6 ± 0.003%) at an AlOxNP concentration of 5 µg/mL. A reduction of the ROS generation in case of the α-phase aluminum oxide nanoparticles correlated well with the data about lower cytotoxicity of these nanoparticles [56].

**Figure 3:**
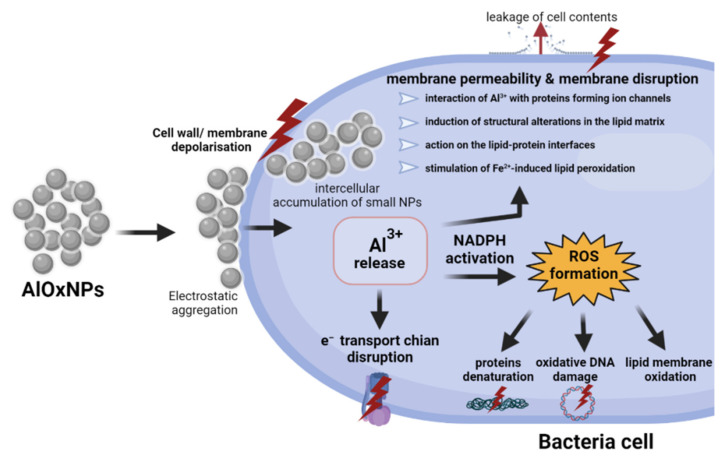
Schematic representation of the main mechanisms of the antibacterial action of AlOxNPs.

The main mechanisms of realization of the bacteriostatic effect of AlOxNPs are the electrostatic interaction of these nanoparticles with the bacterial outer membrane/cell wall and formation of aluminum cations initiating the ROS generation and oxidizing biopolymers. We will consider each of the indicated mechanisms in detail below. Mechanisms reported by the authors are also shown in Figure 3.

**Table 1:** Results of the action of aluminum oxide nanoparticles on the microbial growth reported in the literature.

№	Synthesis Method	Composition	Size, nm and Method	Shape	Concentration	Medium, Conditions	Type of Organism	Bio. Effect	Reference
1	Microwave assisted synthesis using *Prunus* × *yedoensis leaf* extract (PYLE) for recovery	Al_2_O_3,_ different pH	50–100 (FE-SEM)	Sph, hexag	50, 75, 100 μg/mL	NA, 37 °C, 24 h	*S. aureus*, *E. coli*	BS	[19]
2	Commercially available product by Sigma Aldrich (St. Louis, MO, USA; CAS Number 1344-28-1).	Al_2_O_3_	<50 (Supplier’s data) 39 (SEM)	Sph	3, 6, 12, 24, 48, 96, 192 mg/L	-	*Scenedesmus* sp., *Chlorella* sp.	AS	[57]
3	Chemical precipitation using algae extract *L. majucula*	Al_2_O_3_	36.42 (SEM)	Sph	-	30 ± 2 °C, 24–48 h for bacteria; 37 ± 2 °C, 72–96 h for fungi	*S. aureus*, *B. subtilis*, *K. pneumoniae*, *S. paratyphi*, *C. albicans u A. flavus*	BS, FS	[40]
4	Microwave heating using mushroom extract *Colletotrichum* sp.	Al_2_O_3_	39 ± 35 (NTA)	Sph	MIC: 400 ± 1.08 mg/mL for *S. typhi*; 300 ± 2.36 mg/mL for *C. violaceum*; 1000 ± 1.1 mg/mL for *L. monocytogenes*; 250 ± 0.65 mg/mL for *A. flavus*; 150 ± 2.77 mg/mL for *F. oxysporum*	MHA, BHI, 37 °C, 24 h	*S. typhi*, *F. oxysporum*, *A. flavus*, *C. violaceum*, *L.monocytogenes*	BS	[34]
5	Commercially available product by Aldrich (St. Louis, MO, USA; CAS Number 1344-28-1)	Al_2_O_3_	~180 (SEM, DLS)	-	10–1000 μg/L	LB, 30 °C	*E. coli*	BS	[49]
6	Commercially available product by Aldrich (St. Louis, MO, USA)	Chitosan coated Al_2_O_3_- NPs films	<50 (SEM)	Sph	0.05, 0.1 g/mL	MHB, 37 °C, 24 h	*S. aureus*, *P. aeruginosa*, *S. epidermidis*,	BS	[45]
7	Microemulsion method	Al_2_O_3_	30–60 (SEM)	-	MIC: 10 μg/mL	-	*S. typhi*, *V. cholerae*, *K. pneumoniae*	BS	[32]
8	Commercially available product by (Sigma-Aldrich)	Al_2_O_3_	<50 (Supplier’s data)	Sph	0.5 mg/L	26 °C, 16 h	*P. putida*	-	[51]
9	Commercially available product by Aldrich (MERCK, Darmstadt, Germany)	Al_2_O_3_	<100 (TEM)	Rod, irregular, scaly	100 µg/mL	TSB, 37 °C, 24 h	*E. coli*, *S. aureus*, *P. aeruginosa*, *C. albicans*	BS	[54]
10	Commercially available product by Sigma Aldrich (St. Louis, MO, USA; CAS Number 1344-28-1)	Al_2_O_3_	9–182 (HR-TEM, SEM)	Sph	250, 500, 1000, 2000 μg/mL MIC: 1700–3400 μg/mL	LB, 37 °C, 16 h	multidrug-resistant strains of *S. aureus* (MRSA, MSSA, MRCoNS)	BS	[52]
11	Co-precipitation	Al_2_O_3_	35 (SEM)	Irregular sph.	10, 20, 30, 40, 50 mg/mL MIC: 4 mg/mL for *E. coli*; 8 mg/mL for *P. vulgaris*; 6 mg/mL for *S. mutans*, 4 mg/mL for *S. aureus*	NA, SY, BHI, 37 °C, 24 h	*S. aureus*, *S. mutans*, *E. coli*, *P. vulgaris*	BS	[58]
12	Commercially available product (HiMedia Laboratories, India)	Al_2_O_3_	13.5 ± 2.3 (TEM)	Sph	0.25, 0.5, 1 mg/L	NA, NB, 24 h	*P. aeruginosa*, *B. altitudinis*	BS	[59]
13	Commercially available product by Shenzhen Crystal Material Chemical Co., Ltd. (Shenzhen, China)	Al_2_O_3_	40 (SEM)	-	0.05–2.0 g/L	30 °C, 24 h	*B. subtilis*	BC	[60]
14	Solution combustion synthesis	α-Al_2_O_3_	5–30 (HR-TEM, FE-SEM)	flakes-like	5, 500 mg/50 mL; 1000 mg/150 mL	37 °C, 36 h	*K. aerogenes*, *E. coli*, *P. desmolyticum*, *S. aureus*	BS	[36]
15	Microwave assisted synthesis using leaf extracts *Cymbopogon citratus*	Al_2_O_3_	9–180 (HR-TEM); 50 (AFM) 34,5 (DLS)	Sph	1600–3200 µg/mL	MHA, 37 °C, 24 h	multi-drug resistant *P. aeruginosa*	BC	[38]
16	Commercially available product: γ- Al_2_O_3_ Sigma-Aldrich (St. Louis, MO, USA); α-Al_2_O_3_ Sisco Research Laboratories Pvt. Ltd.	α-Al_2_O_3_; γ- Al_2_O_3_	20–30 (α- Al_2_O_3_), 13 (γ- Al_2_O_3_) (Supplier’s data); 280 ± 13 (α- Al_2_O_3_), 256 ± 19 (γ- Al_2_O_3_) (DLS)	-	0.05, 0.5, 1, 5, µg/mL	25 °C, 30 min	*B. licheniformis*	BS	[56]
17	Commercially available product by: γ-Al_2_O_3_ Sigma-Aldrich (St. Louis, MO, USA)	Al_2_O_3_	<50 (Supplier’s data); 51 ± 8, 87 ± 11, 20 ± 13 (NTA)	-	1, 5, 10 g/L	LB, 30 °C, 48 h for *B. cereus*; 37 °C for *P. stutzeri*	*B. cereus*, *P. stutzeri*	-	[48]
18	“Green method” using leaf extract *Cymbopogon citratus*	Al_2_O_3_	34.5 (XRD); 58.5 (HR-TEM)	Sph	0–1500 µg/mL MIC: 250–500 µg/mL for *Candida* spp;	BHI, 28 °C, 48 h	*C. albicans*, *C. parapsilosis*, *C. tropicalis*, *C. glabrata*; fluconazole resistant *C. albicans*, *C. dubliniensis*; fluconazole susceptible *C. albicans*, *C. dubliniensis*	FS	[42]
19	Commercially available product by Aldrich (St. Louis, MO, USA; CAS Number 1344-28-1)	Al_2_O_3_	10–70 (TEM); 78 ± 9 (DLS)	Sph	50, 500, 1000 µg/L	-	*Scenedesmus* sp., *Chlorella* sp.	BS	[61]
20	Commercially available product by Dr. Karl Martin of NovaCentrix, Austin, TX, USA (Product code: M1056, M1049-D; purity: >90%)	Al_2_O_3_	30 & 40 (TEM)	Sph	0.02, 0.04, 0.075, 0.15, 0.30, 0.60, 1.25 and 2.5 mg/plate	NB, 37 °C, 48 h	*S. typhimurium*	-	[62]
21	Gas-phase condensation during laser evaporation of a solid target	Al_2_O_3_	<10 (TEM)	-	0–1 μg/mL	LB, 37 °C, 24–120 h	multi-drug resistant *A. baumanii*	BS	[53]
22	Commercially available product by Sigma-Aldrich (St. Louis, MO, USA; CAS Number 1344-28-1)	Al_2_O_3_	<50 (Supplier’s data); 9–179 (TEM)	Sph	MIC: 1600–3200 μg /mL; MBC: 3200–6400 μg /mL	MHA, 37 °C, 24 h	multidrug-resistant clinical isolates of *E. coli*	BS, BC	[50]
23	Sol–gel synthesis	Chitosan/SiO_2_ nanocomposite with Al_2_O_3_	-	Sph	-	40 °C, 5 h	*S. aureus*, *P. aeruginosa*, *C. albicans*, *A. niger*	BS	[44]
24	Chemical precipitation using *Urtica dioica* as a reducing agent	Al_2_O_3_	10–13 (TEM)	Sph	25, 50, 75 mg/mL	PDM, 25 ± 2 °C, 48 h	*A. niger*, *M. piriformis*	FS	[41]
25	Commercially available (Neutrino Co.)	Al_2_O_3_ coated by chitosan	80 (Supplier’s data)	-	0.025 mg/mL	NB, 37 °C, 24 h	*S. aureus ATCC 6538*	BS	[46]
26	Chemical precipitation	γ-irradiated polyaniline (PANI)/ Al_2_O_3_ NPs composite	17–19 (XDR)	-	17 mg/mL	MHA, 37 °C, 24 h	*E. coli*, *S. aureus*	BS	[37]
27	Chemical synthesis	PANI–Al_2_O_3_ NPs composite	-	-	5, 10 mg/mL	NA, 37 °C, 24 h	*B. subtilis*, *E. coli*	BS	[63]
28	Chemical synthesis, using aluminum waste	Al_2_O_3_	15–50 (XRD)	-	-	MHA, NB, 35 °C, 24–48 h	*E. coli*, *S. typhimurium*, *P. aeruginosa*, *A. aquatilis*, *S. aureus*, *S. pneumonia*, *A. niger*, *A. flavus Penicillium* sp.	BS	[64]
29	Commercially available product by: Sigma-Aldrich, USA (TiO_2_), XIYA REAGENT(Al_2_O_3_)	PLA/Al_2_O_3_ PLA/TiO2 -Al_2_O_3_	21 (TiO_2_), 30 (Al_2_O_3_) (Supplier’s data)	Sph	-	MHA, 37 °C, 24 h	*P. aeruginosa*, *E. coli*	BS	[47]
30	Laser ablation	Al_2_O_3_	10–60 (SEM)	Sph	25, 50, 75, 100 µg/mL	MHA, 37 °C, 24 h	*E. coli*, *P. aeruginosa*, *S. aureus*	BS	[28]
31	Commercially available product by Zhejiang Hongsheng Material Technology Co., China	Al_2_O_3_	60 (Supplier’s data)	Sph	20 mg/L	TSA, 30 °C, 24 h	*B. subtilis*, *E. coli*, *P. fluorescens*	BS	[65]
32	Ball milling method	Al_2_O_3_	100–200 (SEM) 50–60 (XRD)	Sph	MIC: 100µg	NA, 37 °C, 24 h	*B. cereus*, *B. subtilis*, *K. pneumoniea*, *V. cholerae*	BS	[30]
33	Chemical precipitation	γ-Al_2_O_3_ folic acidacid (FA)	23,5 (Al_2_O_3_) & 33 (FA-Al_2_O_3_) (TEM)	Rod	-	-	*P. aeruginosa*, *B. subtilis*	BS	[39]
34	-	Al_2_O_3_–Ag composite	100–200 (TEM)	Sph	1, 10, 30, and 50 wt.%.	LB for *E. coli*, BHI for *S. epidermidis*, 37 °C	*E. coli*, *S. epidermidis*	BS	[43]
35	Commercially available product (Degussa)	Al_2_O_3_	11 (TEM)	Sph	50, 100, 500 mg/L	TSM, 29 °C, for *C. metallidurans*; LB, 37 °C for *E. coli*	*C. metallidurans*, *E. coli*	BC	[66]
36	Laser ablation	Al_2_O_3_ /borosiloxane composite	45 (DLS)	Sph	0.001–0.1 w.%	LB, 37 °C, 24 h	*E. coli*	BS	[29]
37	Commercially available product by Sigma–Aldrich (St. Louis, MO, USA)	Al_2_O_3_	50 (Supplier’s data, TEM)	Rod	1000 mg/L	YEPD, 30 °C, 10 h	*S. cerevisiae*	FS	[67]

BC—bactericidal effect, BS—bacteriostatic effect, AS—algostatic effect, FS—fungistatic effect, Rod—rod-like, Sph—spherical, NA—Nutrient Agar, PDM—potato dextrose medium, MHA—Mueller Hinton Agar, NB—Nutrient broth, TSB—Tryptic soy broth, LB—lysogeny broth, TSM—Tris Salt Mineral medium, YEPD—yeast extract peptone dextrose, BHI—Brain heart infusion, PLA—polylactic acid, NTA—Nanoparticle tracking analysis, DLS—Dynamic light scattering, SEM—Scanning electron microscope, HR-TEM—High-resolution transmission electron microscopy, XRD—X-Ray diffraction analysis.

#### 2.3.1. Electrostatic Interaction between AlOxNPs and Bacterial Cells

It is believed that the positive ζ-potential of AlOxNPs plays an important role in electrostatic adhesion of these nanoparticles on the surface of the bacterial membrane. The negative charge of the bacterial surface is conditioned by the high content of acidic phospholipids and low content of the basic proteins in the composition of the outer membrane of Gram-negative bacteria, as well as the presence of teichoic acids and peptidoglycan in the composition of the cell wall in Gram-positive bacteria [68]. In general, differences in the cell wall structure between Gram-positive and Gram-negative bacteria can affect the interaction between NPs and bacteria. Gram-positive bacteria have the thick outer cell wall formed by a thick peptidoglycan layer with hard polysaccharide chains linked by peptides. The thick outer cell wall can hinder NP penetration into the thick peptidoglycan layer [69]. Multiple studies show that Gram-negative bacteria demonstrate higher sensitivity to the NP impact due to the presence of the outer membrane and thin intermediate peptidoglycan layer [69,70,71].

It is interesting to note that Bhuvaneshwari et al. [59] reported about higher sensitivity of Gram-negative *Pseudomonas aeruginosa* to AlOxNPs compared to Gram-positive *Bacillus altitudinis* upon NP addition even at a low concentration (0.25–1 mg/L).

It was reported that 57%, 36%, and 70% of bacterial cells in *B. subtilis*, *E. coli*, and *P. fluorescens* cultures, respectively, died after 24 h exposure to AlOxNPs. Attachment of nanoparticles to the bacterial surface was shown by TEM. It was assumed that the antibacterial effect was caused by aggregation of nanoparticles with the positive zeta potential on the negatively charged surface of the bacterial cell wall [65].

Extensive attachment of AlOxNPs to the bacterial cell membrane of the multiresistant strain of *P. aeruginosa* led to a significant retardation of the colony growth [38]. It was also established in other studies that AlOxNP aggregation on the bacterial cell surface is one of the key mechanisms of the antibacterial activity. Flocculation of nanoparticles on the bacterial surface was observed, which compromised the integrity of the cell wall and membrane of Gram-positive multiresistant *S. aureus* [52], Gram-negative *E. coli*, and *C. metallidurans* [50,66] as well as *A. baumanii* [53]. It was shown by scanning confocal microscopy that the bacterial cell wall changed its morphology after an impact of positively charged nanoparticles, which also confirms the fact of integrity loss in the bacterial cell wall and membrane with the following penetration of nanoparticles inside a cell [72].

After AlOxNP application, Muzammil et al. [53] found bacterial biopolymers in the intercellular environment due to bacterial membrane damage and subsequent leakage of the bacterial cell content of *A. baumanii*. Mu et al. [60] also revealed extensive electrostatic attachment of AlOxNPs on the surface of *Bacillus subtilis*; consequently, it was proposed to use these nanoparticles to remove *B. subtilis* from a fermentation broth [60]. Ansari et al. reported about inhibition of the colony growth of *E. coli* clinical isolates due to multiple, extensive, AlOxNP-induced injuries of cell membranes [50]. This observation was confirmed in another study using *E. coli* as test-bacteria, which showed a significant decrease in the viability of *E. coli* cells upon 24 h treatment with AlOxNPs [66]. Using TEM, it was also established that AlOxNPs of a lower size were uniformly distributed inside bacterial cells, while agglomerates of larger sizes remained to be attached to the surface of the cell membrane. Fourier-transform infrared spectroscopy (FTIR) confirmed an interaction of AlOxNPs (<50 nm) with molecules being constituents of the outer membrane of *E. coli*: phosphatidylethanolamine and lipopolysaccharides [50].

#### 2.3.2. ROS-Release

Another mechanism of an AlOxNP impact on the growth of bacterial cultures is induction of ROS formation, mediated by generation of aluminum cations in a solution. An increase in the ROS intracellular level in bacteria *C. metallidurans* and *E. coli* was revealed after two hours of exposure to AlOxNPs [66]. It was found that release of Al^3+^ ions upon mixing AlOxNPs in water was 13, 17, and 20 µg/L at NPs concentrations of 0.25, 0.5, and 1 mg/L, respectively [59]. Mukherjee et al. [73] compared the antibacterial effect of both AlOxNPs in different concentrations and solutions containing the equivalent concentration of aluminum oxide ions. A similar degree of the antibacterial activity was found for AlOxNPs and the equivalent concentration of the aluminum salt [73], which also confirms a contribution by generation of aluminum free ions to damage of bacterial cells. An increased Al^3+^ concentration can stimulate ROS generation due to membrane depolarization as well as activation of enzyme NADPH oxidase in cells [74]. There are also reports about permeabilization of the *E. coli* membrane upon an action of aluminum ions, which subsequently facilitated transport of toxic ions of other metals, including iron, enhancing the antibacterial effect [75]. Interaction of Al^3+^ with phospholipids of the cell membrane induces a range of its structural and functional disorders. Such disorders include the direct interaction of Al^3+^ with proteins generating ion channels, receptors, and enzymes; induction of structural changes in the lipid membrane; and the activity at the lipid/protein interface [76]. In general, ability of Al^3+^ ions to enhance oxidative damage of membranes is a well-known phenomenon. It is known that aluminum ions accelerate peroxidation of membrane lipids induced by iron (II) ions upon acidic values of pH [77,78].

### 2.4. Genotoxic Action of AlOxNPs

Several works demonstrated the genotoxic effect of AlOxNPs. In particular, significant (*p* < 0.05) DNA damage was found upon treatment of *P. aeruginosa* and *B. altitudinis* cells [59]. It was reported earlier that oxidative stress induced by nanoparticles can act as the main factor of DNA damage in bacterial cells [79]. Formed ROS cause DNA chain breakage, removal of nucleotides [15], DNA-protein crosslinks [80], modifications of nucleotide bases [81], and deoxyribose oxidation by addition of ^•^OH radicals to double bonds.

### 2.5. AlOxNP Action on Microalgae of Water Reservoirs

Due to wide application of aluminum oxide nanoparticles in the industry, investigation of the toxic effect of these nanomaterials on aqueous ecosystems upon their penetration into water reservoirs is of great interest. The number of reports about AlOxNP toxicity to microscopic algae is increasing. Sadiq et al. revealed the toxic effect of AlOxNPs on *Scenedesmus* sp. and *Chlorella* sp. obtained from the open water reservoir. The half maximal effective concentration (EC50) was 39.35 mg/L for *Scenedesmus* sp. and 45.4 mg/L for *Chlorella* sp. 72 h after introduction of nanoparticles [57]. Moreover, Pakrashi et al. [61] in long-term experiments in artificial water reservoirs (microcosm) noted the short-term (5 days) effect of AlOxNPs on the resident population of algae *Scenedesmus* sp. and *Chlorella* sp., accompanied by a sharp decrease in the viability of algae cells by about 25%. Upon long-term impact over 7 months (210 days), a gradual restoration of viability indicators was shown [61].

### 2.6. Antimycotic Effect of AlOxNPs

Investigation of an AlOxNP effect on the growth of microscopic fungi has attracted considerable interest. Several studies revealed a NP effect not only on bacterial cells but also on microscopic fungi. For example, AlOxNP introduction inhibited the growth of fungi *C. albicans* and *A. flavus* [40], *A. niger* and *M. piriformis* [41]. AlOxNP application facilitated destruction of membranes in yeast *Saccharomyces cerevisiae* only in high concentrations (more than 1000 µg/mL) [67]. Inhibition of the growth of *Aspergillus niger*, *Aspergillus flavus*, and *Penicillium* sp. enhanced with an increase in the nanoparticle concentration was confirmed in the recent study [64]. AlOxNPs inhibited the growth of fluconazole-sensitive and resistant *C. albicans* and *C. dubliniensis*. It was revealed using electronic microscopy that AlOxNPs not only adhere to the surface but also penetrate inside fungal cells of the genus *Candida*, lead to their morphological disorders, and inhibit their physiological activity, which finally results in cell death [42]. The obtained information allows suggesting a possibility to use these nanoparticles as antifungal agents.

### 2.7. Cytotoxicity of AlOxNPs

The question about cytotoxicity of nano-aluminum oxide against eukaryotic cells has also aroused considerable interest and is quite controversial. On the one hand, several studies showed that AlOxNPs exhibited low toxicity to eukaryotic cells in in vitro experiments. For example, it was demonstrated that there was no effect on viability of the HeLa cell line when adding AlOxNPs at a concentration of 120 µg/mL; morphological changes in cells were observed when a concentration of 240 µg/mL was used [53]. Moreover, penetration (10–200 µg/mL) through membranes of L929 and BJ cells without a significant reduction in the viability level and changes in the level of cell apoptosis was found after 24 h exposition [14]. On the other hand, a decrease in the viability of A549 cells (human lung carcinoma) was noticed when AlOxNPs were added at concentrations of 10 and 25 µg/mL after 24 h of exposure. It was assumed that this effect was conditioned by cell membrane depolarization [82]. In addition, it was revealed that AlOxNPs affect the growth and development of four cell lines: VERO, HEp-2, A549, and MDA-MB-231. The LD_50_ values for VERO and HEp-2 cells were 31.25 µg/mL, for A549 and MDA-MB-231 5.625 µg/mL [32]. It is interesting to note AlOxNP cytotoxicity to nerve cells. As it is known, neurons are more sensitive to external impacts and are distinguished by low resistance to stress factors in in vitro experiments. In particular, it was shown that AlOxNP introduction caused the neurotoxic effect in vitro conditioned by the development of the oxidative stress in nerve cells with the characteristic increase in the level of lactate dehydrogenase expression, disorder of the mitochondrial function, disruption of the cell cycle, and induction of apoptosis [83]. Another study also confirmed the ROS-induced peroxidation of lipids and proteins, glutathione depletion, and mitochondrial dysfunction of brain tissue cells in rats upon chronic exposure to AlOxNPs during 28 days [84]. A special place belongs to the studies devoted to investigation of the role of aluminum in the development of the neurodegenerative diseases of the central nervous system including Alzheimer’s disease [85] and Parkinson’s disease [86]. A relationship between the process of aluminum accumulation in the body’s tissues including the brain tissue [87], aggregation of amyloid β (Aβ) [88], development of the neuro-inflammatory response [89], and the pathogenesis of Alzheimer’s disease is a subject of many studies carried out over the last decades. There are several assumptions and evidence confirming aluminum-induced neurotoxicity; however, the strict mechanism of aluminum neurotoxicity is still open to further discussion [85].

## 3. Conclusions

A search for new methods of controlling antibiotic resistant bacterial infections is an important problem for public health worldwide. Therefore, the use of non-organic nanomaterials, mainly metal and metal oxide nanoparticles as antibacterial agents of the new generation, is considered. On the background of the proved antibacterial effectiveness of metal oxide nanoparticles with known clear mechanisms of action on bacteria (titanium dioxide, iron oxide, silver oxide, and zinc oxide), aluminum nano-oxide remains a poorly explored material. Despite wide distribution of aluminum in nature and the wide application of AlOxNPs in production, the use of these nanoparticles in biomedical applications including the antibacterial purpose is hampered. This is determined by the poor evidence base of the effectiveness of AlOxNP influence on the bacterial growth as well as the low reactivity of aluminum oxide. Nevertheless, several successful studies of AlOxNPs carried out in recent years including on multiresistant and clinical bacterial strains give encouraging results. It is known that the antibacterial effect of AlOxNPs is manifested, as a rule, only at high concentrations of nanoparticles and is determined by adsorption of these nanoparticles on the bacterial surface, as well as the development of aluminum cations that facilitate ROS generation causing oxidation of biomacromolecules and leading to the death of bacterial cells. The reported fungistatic activity of AlOxNPs against several fungal species is also of great interest and requires more detailed consideration. Aluminum oxide nanoparticles have low toxicity and do not induce apoptosis in eukaryotic animal cells [14,15]; however, the ability of aluminum to induce oxidative stress and the neurotoxicity demonstrated in numerous studies [90], as well as its role in the development of neuropathologies [91], indicate the need for a deeper investigation and further study of the mechanisms of its impact on biological systems.

## Data Availability

The raw data supporting the conclusions of this article will be made available by the authors, without undue reservation.
